# The Effectiveness of Myo-Inositol in Women With Polycystic Ovary Syndrome: A Prospective Clinical Study

**DOI:** 10.7759/cureus.53951

**Published:** 2024-02-10

**Authors:** Minthami Sharon P, Mellonie P, Anu Manivannan, Priyanka Thangaraj, Logeswari B M

**Affiliations:** 1 Department of Obstetrics and Gynaecology, Sree Balaji Medical College and Hospital, Chennai, IND; 2 Department of Pathology, G. R. Medical College, Mangalore, IND; 3 Reproductive Medicine and Surgery, Sri Ramachandra Institute of Higher Education and Research, Chennai, IND

**Keywords:** luteinizing hormone, follicle-stimulating hormone, polycystic ovarian syndrome, metabolic derangements, insulin resistance, myo-inositol

## Abstract

Background

Polycystic ovarian syndrome (PCOS) is a multifaceted complex endocrine disorder showing an alarming rise in women worldwide. Insulin resistance is the chief driving force in the pathogenesis of PCOS. Myo-inositol is an upcoming insulin-sensitizing agent, which is a second messenger responsible for insulin-mediated intracellular glucose transport. This study aims to evaluate the efficacy of myo-inositol and its clinical, hormonal, and metabolic profile in treating women with PCOS.

Methodology

A prospective clinical study was conducted over 18 months in the Department of Obstetrics and Gynecology at Sree Balaji Medical College and Hospital, Chennai, after obtaining permission from the Institutional Ethical Committee. A total of 90 women diagnosed with PCOS, according to Rotterdam’s criteria, were included in the study. They received tablet myo-inositol 1 g BD for six months. Before the start of the therapy, detailed history and baseline investigations were recorded and subsequently re-assessed at the end of six months.

Results

Around 68% of patients restored menstrual cycle regularity. There was a statistically significant decrease in luteinizing hormone (LH) (10.31 ± 7.92 to 7.42 ± 6.25; p = 0.002), LH/follicle-stimulating hormone ratio (2.34 ± 0.34 to 1.91 ± 0.32; p = 0.000), fasting serum insulin levels (16.71 ± 13.92 to 13.18 ± 9.41; p = 0.041), and homeostatic model assessment for insulin resistance (4.52 ± 1.34 to 2.74 ± 1.28; p = 0.041).

Conclusions

According to our study, it was observed that myo-inositol led to a statistically significant improvement in the hormonal and metabolic profile of PCOS patients. Moreover, it is safe and has good compliance. Hence, we can justify the addition of myo-inositol to the armamentarium for PCOS management.

## Introduction

Polycystic ovary syndrome (PCOS), the mounting syndrome, is currently recognized as a multifaceted genetically complex endocrine disorder, with an alarming increase in prevalence among women of all age groups. The association of amenorrhea with bilateral polycystic ovaries and obesity was first described in 1935 by Stein and Leventhal [1], since then PCOS has been a “gynecological curiosity with multisystem endocrinopathy” [2]. Over 2.2% to 26% [3] of women are affected globally, with up to 70% of women remaining undiagnosed. In north India, the prevalence is estimated to be 3.7% [4], with 9.13% to 36% prevalence in adolescent girls according to a 2012 study [5]. Regardless of age, it has a significant and wide range of clinical implications. The spectrum includes reproductive dysfunction (menstrual irregularities, normogonadotropic anovulation-WHO II cohort, hirsutism, infertility, and pregnancy complications), metabolic dysregulation (insulin resistance, impaired glucose tolerance, type 2 diabetes, dyslipidemia, and metabolic syndrome), cardiovascular risk factors, and psychological implications (anxiety, depression, negative body image, and worsened quality of life) [6,7].

Insulin resistance plays a key role in the clinical development of PCOS. Insulin resistance is observed in 70-80% of obese PCOS women and 20-25% of lean PCOS women [8]. The overall prevalence of insulin resistance in PCOS ranges between 50% and 75% [9,10]. The first-line intervention in the management of PCOS is lifestyle modifications, including dietary changes and increased physical activity. Energy-deficit diet and correcting the macro and micronutrient composition of the diet improves insulin sensitivity regardless of weight loss [11]. Myo-inositol (C_6_H_12_O_6_, hexahydroxy cyclohexane) has been newly recognized and has drawn interest for further studies in the management of PCOS and is naturally available [12]. It is commonly found in fruits (grapefruit), vegetables, beans, almonds, and walnuts. It forms an important component of membrane lipids. Various biological molecules such as inositol phosphates (IP3), phosphatidylinositol phosphate lipids (PIP2/PIP3), and inositol glycans (IPGs) which act as second messengers for numerous biological activities contain myo-inositol as a component [12]. Hence, it plays a key role in the efficient functioning of various cellular functions such as smooth muscles, osteogenesis, regeneration and conduction of peripheral nerves, and reproduction [13-18]. Myo-inositol is similar to insulin in its action. It helps in the uptake of glucose in the cells through intracellular insulin uptake pathways, thereby increasing insulin sensitivity and decreasing insulin resistance which is the cornerstone in the development of various metabolic dysfunctions in PCOS [12,19]. Inositol is the precursor for PI3-kinase. It has been proposed that the deficiency of inositols plays a key role in the development of insulin resistance in PCOS. It has been observed that there is an increased urinary excretion of inositol in patients with PCOS leading to its deficiency [20]. The above observations and evidence have gained momentum and thrown light on myo-inositol as a potential insulin-sensitizing agent to be utilized as a treatment option in PCOS patients to restore metabolic function and consequently ovulation. At present, only limited studies are available from India. This study aims to determine the effect of myo-inositol in the treatment of PCOS (clinical, hormonal, and metabolic). The primary objectives are to assess the effect of myo-inositol on its clinical improvement by assessing the restoration of regular menstrual cycles, hormonal parameters (follicle-stimulating hormone (FSH), luteinizing hormone (LH), and LH/FSH ratio), and metabolic parameters (fasting blood sugar (FBS), serum fasting insulin, and homeostatic model assessment of insulin resistance (HOMA-IR) index). The secondary objective is to observe any side effects of the drug.

## Materials and methods

**Figure 1:**
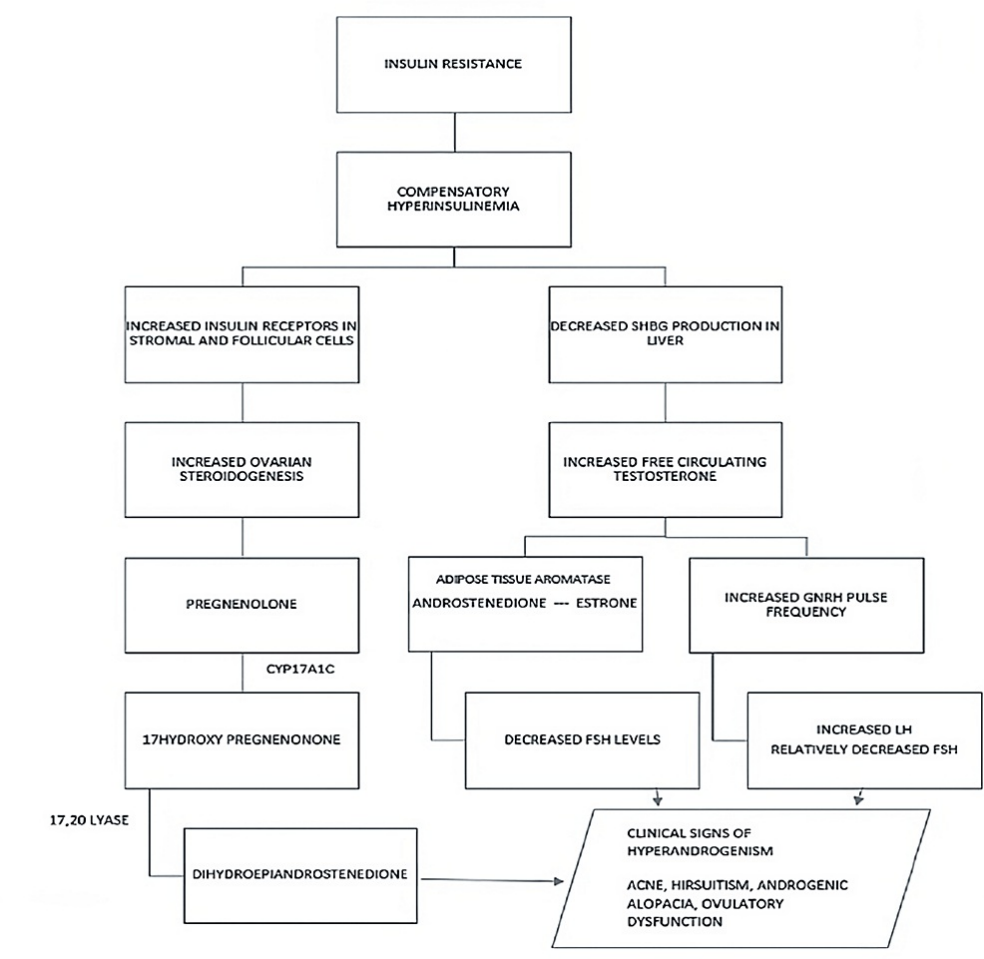
Flowchart showing the effect of insulin resistance in the development of PCOS symptoms. The figure is made by the corresponding author. PCOS = polycystic ovary syndrome; SBHG = sex hormone-binding globulin; GNRH = gonadotropin hormone-releasing hormone; LH = luteinizing hormone; FSH = follicule-stimulating hormone

This prospective clinical study was conducted in the outpatient department under the Department of Obstetrics and Gynaecology (OBG) in a tertiary care hospital in Chennai over 18 months. The interaction of various factors such as Insulin resistance along with hyperandrogenism, altered gonadotropins, and target genes constitutes the pathophysiology of PCOS ( Figure 1).

Inclusion criteria included women aged 18-40 years who presented with PCOS as per the Rotterdam criteria (ESHRE/ASRM 2003) [21] and agreed to participate in the study after providing written informed consent. The diagnosis of PCOS was made based on at least two of the three major criteria, namely, oligo/anovulation, clinical and/or biochemical signs of hyperandrogenism, and polycystic appearance in one or both ovaries on ultrasonography (an ovarian volume >10 mL^3^ and/or >12 follicles measuring 2-9 mm in at least one ovary). The exclusion criteria were known cases of PCOS on treatment (e.g., oral contraceptive pills), patients with significant cardiac, pulmonary, renal, hepatic, neurological, psychiatric illness and malignant disease, hyperprolactinemia, adrenal disorders (congenital adrenal hyperplasia), thyroid disorders, pregnant/lactating women, and those with type 1/2 diabetes mellitus [22].

The study was performed after obtaining clearance from the Institutional Human Ethics Committee (IHEC), Bharat Institute of Higher Education and Research, under which Sree Balaji Medical College functions (reference number: 002/SBMC/IHEC/2017/950). A total of 100 consecutive women were recruited to receive a tablet myo-inositol 1 g twice daily continuously for six months. Before starting therapy, a detailed history was taken, clinical assessment and baseline investigations were done, namely, serum FSH and LH (done on day two or three of a spontaneous or induced menstrual cycle), FBS, fasting serum insulin, and related parameters such as LH/FSH ratio and HOMA-IR were calculated. Cases were reassessed at the end of six months for regularity of the menstrual cycle. All baseline investigations were repeated and side effects, if any, were recorded [22,23].

Statistical analysis

The obtained data were analyzed statistically using SPSS software version 21 (IBM Corp., Armonk, NY, USA). Continuous variables were summarized as mean with standard deviations or median with interquartile range. Categorical variables were summarized as frequency with percentages. Student’s paired t-test was used to compare continuous variables, and a p-value <0.05 was considered statistically significant.

## Results

Of the 100 women, only 90 (90%) completed the study, and the other 10 (10%) were lost on follow-up. Hence, statistical analysis was done only for 90 women. The baseline characteristics of the study participants are presented in Table 1.

**Table 1:** Baseline characteristics of the study population before commencing treatment (N = 90). FSH = follicle-stimulating hormone; LH = luteinizing hormone; FBS = fasting blood sugar; HOMA-IR = homeostatic model assessment for insulin resistance

Characteristics	N (%)/Mean ± SD
Age (in years)	26.70 ± 6.78
Menstrual irregularity	88 (98)
Marital status - unmarried	55 (61)
FSH (mIU/mL)	5.33 ± 0.85
LH (mIU/mL)	10.31 ±7.92
LH/FSH	2.34 ± 0.34
FBS (mg/dL)	83.33 ± 8.84
Fasting serum insulin (µIU/mL)	16.71 ± 13.92
HOMA-IR	4.52 ± 1.34

The mean ± SD age of patients was 26.70 ± 6.78 years. Around 55 (61%) patients were unmarried. About 38 (42%) participants had infrequent cycles with scanty flow before the start of the study. About 32 (36%) participants had infrequent cycles and normal flow. Amenorrhoea was recorded in 14 (15%) patients before the onset of therapy. Almost 62 (69%) patients restored menstrual cycle regularity. Among amenorrhoeic patients, 11 out of 14 (79%) had a spontaneous restoration of menses (Table 2).

**Table 2:** Different types of menstrual dysfunction among the study participants (N = 90). Values are represented as mean ± SD and frequency N (%). FIGO = International Federation of Gynaecology and Obstetrics

Menstrual dysfunction (according to FIGO)	Number of patients	Percentage (%)
Infrequent cycles with scanty flow (>38 days with patient-determined light flow volume)	38	42
Infrequent cycles (>38 days)	32	36
Amenorrhea	14	15
Scanty flow (patient determined)	3	3
Frequent cycles (<24 days)	2	2
Regular cycles (≥24 days to ≤38 days)	2	2
Total	90	100

The primary outcomes were the clinical parameters described earlier including hormonal parameters such as FSH, LH, and LH/FSH, as well as metabolic parameters including FBS, fasting insulin, and HOMA-IR. FSH was measured both at baseline (5.33 ± 0.85) and following treatment (5.59 ± 0.97). This fall in FSH was not significant statistically. The baseline LH was 10.31 ± 7.92, and after six months of therapy, LH reduced to 7.42 ± 6.25, which was statistically significant. Thus, the LH/FSH also normalized after six months of myo-inositol therapy in our study. The metabolic profile also showed significant improvement after six months of therapy with myo-inositol (Table 3). FBS was measured pretreatment (83.33 ± 8.84) and post-treatment (82.71 ± 8.60). Although the difference in the reduction of FBS was not statistically significant, there was a significant (p = 0.041) reduction in the fasting insulin levels in the blood. Moreover, the HOMA-IR pretreatment and post-treatment reduced significantly (p = 0.048).

**Table 3:** Effect of myo-inositol on hormonal and metabolic parameters (N = 90). Values are presented as mean ±SD. Paired t-test was done, and p-values <0.05 are significant. FSH = follicle-stimulating hormone; LH = luteinizing hormone; FBS = fasting blood sugar; HOMA-IR = homeostatic model assessment for insulin resistance

	Pretreatment (mean ± SD)	Post-treatment (mean ± SD)	P-value
Hormonal parameters			
FSH (mIU/mL)	5.33 ± 0.85	5.59 ± 0.97	0.412
LH (mIU/mL)	10.31 ± 7.92	7.42 ± 6.25	0.002
LH/FSH	2.34 ± 0.34	1.19 ± 0.32	<0.001
Metabolic parameters			
FBS (mg/dL)	83.33 ± 8.84	82.71 ± 8.60	0.085
Fasting serum insulin (µU/mL)	16.71 ± 13.92	13.18 ± 9.41	0.041
HOMA-IR	4.52 ± 1.34	2.74 ± 1.28	0.048

**Figure 2:**
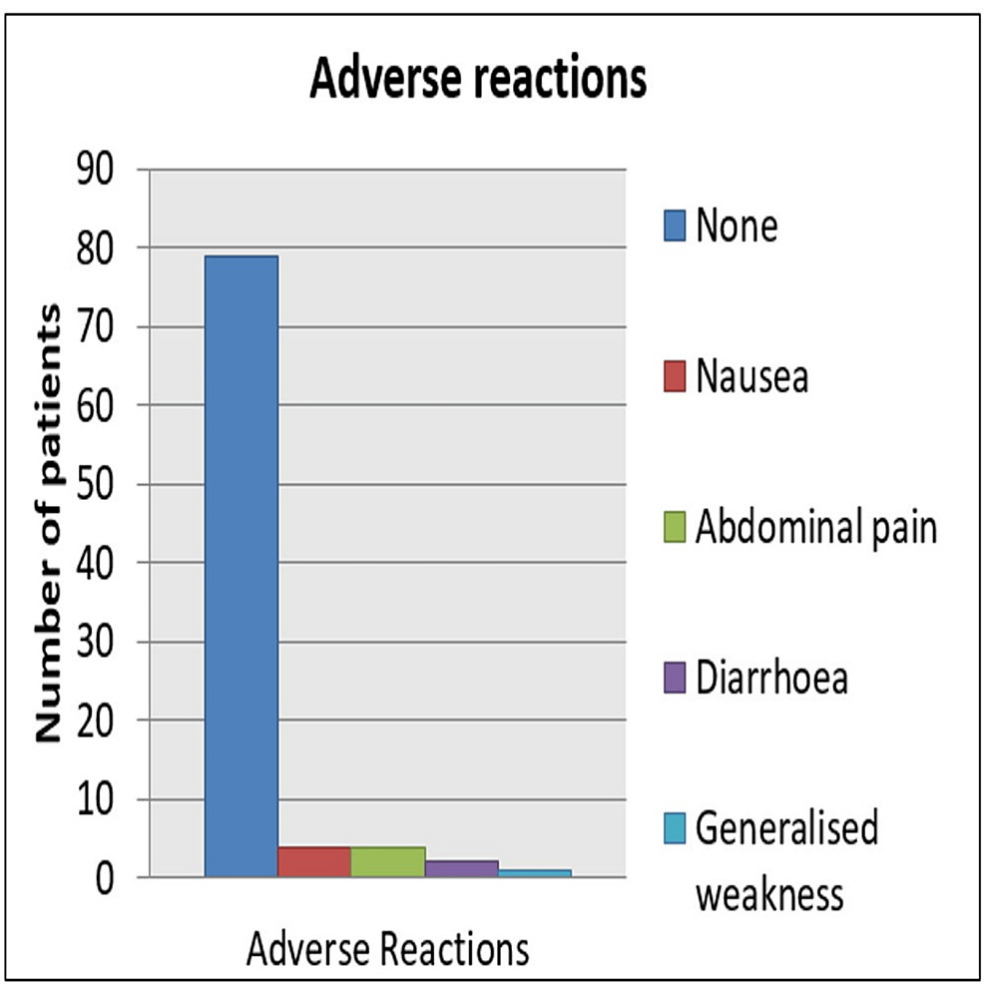
Adverse drug reactions with myo-inositol.

Overall, 85% of patients (79/90) did not have any adverse drug effects. Few complained of nausea, abdominal pain, diarrhea, and generalized weakness (Figure 2).

## Discussion

PCOS is a complex syndrome associated with androgen excess and insulin resistance. Myo-inositol helps reduce androgen and insulin resistance. Hence, based on the current evidence, PCOS with insulin resistance can be treated with myo-inositol. Myo-inositol also impacts the hypothalamo-pituitary-ovarian axis and reduces the circulating estrogen levels in the blood. Myo-inositol may be considered a cheaper and safer alternative for treating PCOS. Our study showed a significant decrease in the FSH, LH, and HOMA-IR which is consistent with the previous studies. Moreover, regularization of the menstrual cycle was noted by the end of six months similar to other studies [24]. Although similar results have been reported in another study, a significant increase in pregnancy rate has not been found compared to other drugs [25].

In 2021, Merviel et al. conducted a review of the literature regarding the impact of myo-inositol on the reproductive outcome of PCOS women undergoing assisted reproductive techniques and found that myo-inositol is effective in restoring ovarian function as well as improving the oocyte and embryo quality [26]. Artini et al. in a clinical trial conducted in 2013 with 50 patients concluded that, after 12 weeks of therapy with myo-inositol, there was a significant reduction in plasma LH, prolactin, insulin levels, and LH/FSH. Insulin resistance measured as glucose-to-insulin ratio and HOMA-IR were also significantly reduced. Almost all women regained their regularity of menstrual cycles whereas there was no change in those treated with placebo [27]. In another double-blind study among 42 patients, Constantino et al. in 2003 found that there was a significant decrease in circulating free testosterone and free insulin levels, as well as a decrease in triglycerides and systolic and diastolic blood pressure. It was also observed that the majority of women regained regular menstrual cycles and spontaneous ovulation compared to the placebo group [28]. In 2008, Genazzani et al. studied 20 overweight women with PCOS and found a significant reduction in FSH, LH, LH/FSH, prolactin, fasting insulin, and HOMA-IR. Moreover, they found a restoration of menstrual and reproductive functions after 12 weeks of therapy with myo-inositol [29]. In 2016, Ozay et al. conducted a comparative study between combined oral contraceptives and myo-inositol and concluded that myo-inositol is superior to combined oral contraceptives in lowering androgen levels. It also has a positive effect on ovarian volume and anti-Mullerian hormone) [30].

Safety of myo-inositol

In a study by Carlomagno et al., mild gastrointestinal side effects such as nausea, flatulence, and diarrhoea are seen in patients treated with high doses of myo-inositol (12 g/day) [24], similar to our study. Thus, myo-inositol is a safe drug with better patient compliance, which, in turn, leads to better therapeutic outcomes in PCOS patients. It is beneficial during pregnancy and has been studied for primary prevention of neural tube defects and gestational diabetes mellitus [31]. Myo-inositol is a promising drug with low adverse reactions and better hormonal and biochemical reversal of PCOS. Moreover, three months of administration of myo-inositol before ovulation induction has shown good follicular response and lesser estradiol levels on the day of ovulation trigger thereby reducing the risk of ovarian hyperstimulation syndrome. It is also found to improve oocyte and embryo quality [32]. These findings in previous studies opened a huge scope for further research about this fascinating molecule, myo-inositol.

Study limitations

A larger sample size and longer observation period are needed to evaluate the long-term effects of myo-inositol and how it affects the metabolic profile of patients to explore its adverse effects. Effects on different phenotypes of PCOS, cutaneous manifestations of hyperandrogenism, body mass index, free androgen index, and lipid profile were not monitored.

## Conclusions

PCOS is a major metabolic and hormonal dysfunction that often remains undiagnosed. Lifestyle modification would be the sheath anchor in the treatment of PCOS. Myo-inositol is a new drug that is gaining importance in the treatment of PCOS. Our study used a comprehensive approach to show that treatment with myo-inositol resulted in the regularization of menses and improvement in endocrinological (significantly lower LH and LH/FSH) and metabolic parameters (significantly lower fasting insulin and HOMA-IR). Moreover, it caused only minor adverse effects. Thus, the present study justifies the use of myo-inositol as a safe and effective alternative and a prospective molecule to be added to the armamentarium of PCOS treatment. Further studies on the cost-effectiveness of myo-inositol and its widespread use can be explored in the future.
